# Will drug resistance against dolutegravir in initial therapy ever occur?

**DOI:** 10.3389/fphar.2015.00090

**Published:** 2015-04-29

**Authors:** Mark A. Wainberg, Ying-Shan Han

**Affiliations:** Lady Davis Institute for Medical Research, McGill University AIDS Centre, Jewish General HospitalMontreal, QC, Canada

**Keywords:** antiretroviral, drug resistance, HIV, integrase inhibitors

## Abstract

Dolutegravir (DTG) is a second-generation integrase strand transfer inhibitor (INSTI) and INSTIs are the latest class of potent anti-HIV drugs. Compared to the first generation INSTIs, raltegravir, and elvitegravir, DTG shows a limited cross-resistance profile. More interestingly, clinical resistance mutations to DTG in treatment-naive patents have not been observed to this date. This review summarizes recent studies on resistance mutations to DTG and on our understanding of the mechanisms of resistance to DTG as well as future directions for research.

## Introduction

Antiretroviral therapy (ART) usually involves a combination of at least three different antiretroviral (ARV) drugs to maximally suppress HIV propagation and stop the progression of HIV disease. ART has led to a huge decrease in HIV-related morbidity and mortality, particularly when it is used in early stages of disease. Currently, 29 ARV drugs, belonging to six different classes that act at different stages of the HIV life cycle, have been approved by the Food and Drug Administration (FDA) of the U.S. for use in the clinic. However, HIV can develop resistance to evade the effects of treatment by almost all classes of ARV drugs.

Integrase strand transfer inhibitors (INSTIs), which block the integration of HIV viral DNA into host chromosomal DNA, are the latest class of ARV drugs. So far, there are three approved INSTIs, raltegravir (RAL), elvitegravir (EVG), and dolutegravir (DTG). RAL and EVG, the first generation INSTIs, are highly effective in treatment of patients with HIV. But HIV resistance to both RAL and EVG can emerge relatively rapidly in tissue culture and can also arise in patients as a result of single mutations or primary mutations that are in combination with a secondary mutation in integrase (Geretti et al., 2012; Mesplede et al., 2013; Quashie et al., 2013b; Grobler and Hazuda, 2014). Cross resistance to DTG between RAL and EVG has also been documented.

However, DTG, a second generation INSTI, has shown a superior barrier to resistance compared to that seen with RAL or EVG. DTG is able to inhibit RAL- and EVG-resistant viruses either completely or partially *in vitro*. Importantly DTG is the only anti-HIV drug that HIV has not developed resistance mutations against when the drug is used in first line therapy (Mesplede and Wainberg, 2014). This review summarizes recent findings on resistance mutations to DTG and the underlying mechanisms that may be responsible for the absence of resistance to DTG in first line therapy.

## HIV Resistance to DTG

Raltegravir and EVG share similar resistance profiles in regard to mutations in the active site of integrase. Furthermore, resistance to RAL and EVG can emerge rapidly both *in vitro* and in patients (Geretti et al., 2012). Resistance to RAL includes three major mutational pathways involving positions Y143, Q148, and N155 in integrase. For EVG, major primary mutations include T66I, E92Q, N155H, and Q148H/K/R. Extensive cross resistance between RAL and EVG can occur through mutations at position 155 and 148. In comparison with the first generation INSTIs RAL and EVG, DTG has a higher resistance barrier (Wainberg et al., 2013; Llibre et al., 2014; White et al., 2014), and also shows less sensitive to the changes caused by mutations at N155 and Q148 (Abram et al., 2013; Quashie et al., 2013b; **Table 1**).

**Table 1:** Major resistance pathways to raltegravir (RAL), elvitegravir (EVG), and dolutegravir (DTG).

	Mutational pathways	Fold resistance		
		RAL	EVG	DTG
Y143 pathway	Y143C Y143R T97A/Y143C T97A/Y143R L74M/T97A/Y143G L74M/T97A/E138A/Y143C	<10 <50 >100 >100 <50 <20	<2 <2 <2 <2 ND ND	<2 <2 <2 <2 <2 <2
N155 pathway	N155N E92Q/N155H L74M/N155H	<50 <100 <50	50 >100 <50	<2 <10 <2
Q148 pathway	Q148H Q148K Q148R E138K/Q148H E138K/Q148K E138K/Q148R G140S/Q148H G140S/Q148K G140S/Q148R E138A/G140S/Y143H/Q148H	<20 <100 <50 <10 >100 >100 >100 <10 >100 >100	<10 <100 <100 <20 >100 >100 >100 <100 >100 ND	<2 <2 <2 <2 <10 <10 <20 <2 <10 <50
R263K pathway	R263K R263K/H51Y	<1 3–5	3 3	4 4–6
G118R pathway	G118R G118R/H51Y G118R/E138K	10–17 ND 4–20	>5 ND 4–5	>8 ND 8–13

*ND, not detected. Quashie et al. (2013b, 2014).*

Some mutations that are potentially involved in integrase resistance to DTG and that were selected *in vitro* and *in vivo* include F121, S153, G118, E138, and R263 (Kobayashi et al., 2011; Quashie et al., 2012). These mutations alone or in combination with accessory mutations did not abrogate susceptibility to DTG, but did impair viral replicative fitness to varying extent (**Table 2**). The most common mutation identified in cell culture selections with DTG was R263K and this substitution was shown to confer low-level resistance to DTG (fold change, FC = 2.3-fold; Quashie et al., 2012). R263K also impaired strand transfer activity and decreased viral replication capacity (RC). R263K has been reported in several treatment-experienced, INSTI-naïve patients (Cahn et al., 2013).

**Table 2:** Effects of mutations in integrase on resistance to INSTIs, viral replication capacity (RC), and strand transfer activity.

Genotype	Strain	Phenotypic assay	Susceptibility to INSTIs (Fold change)			RC (FC)	STA (FC)	Reference
			RAL	EVG	DTG			
WT	NL43	TZM-bl cells	1	1	1	1	1	Wares et al. (2014)
M50I			0.47	5.45	1.94	0.92	1.1	
R263K			1.85	21.4	8.55	0.7	0.22	
M50I/R263K			3.56	34.44	15.59	0.7	0.31	
WT	NL43	PhenoSense	1	1	1	1	1	Mesplede et al. (2013)
H51Y			1.11	2.06	1.25	0.89	1.07	
R263K			1.21	3.28	1.95	0.7	0.45	
H51Y/R263K			2.94	41.5	6.95	0.11	0.2	
WT	NL43	TZM-bl cells	1	1	1	1	1	Mesplede et al. (2014)
E138K			1	0.8	0.4	0.83	2.4	
R263K			1.2	21.8	2.3	0.72	0.5	
E138K/R263K			1	16	4.3	0.71	0.6	
WT	NL43	PM1 cells	1	1	1	1	1	Bar-Magen et al. (2010), Quashie et al. (2013a)
G118R			0.78	1	–	0.02	0.04	
G118R/H51Y			–	–	–	–	0.06	
G118R/E138K			2.33	2	–	0.13	0.08	

*RC, replication capacity; STA, strand transfer activity; WT, wild-type.*

Usually a combination of primary mutation and secondary mutations increase levels of drug resistance while restoring viral replication fitness. In cell culture selections in the presence of DTG, R263K was often observed together with H51Y in subtype B and recombinant A/G viruses. H51Y alone has no effects on resistance to DTG. However, the addition of H51Y to R263K increases resistance to DTG to ∼eightfold in a HIV susceptibility assay using TZM-bl cells and dramatically decreases viral RC by ∼90%; enzyme strand transfer activity is reduced by ∼80% (Mesplede et al., 2013).

Similarly, M50I was identified as an accessory mutation in association with R263K in cell cultures selected under DTG pressure and has been also reported in a patient failing treatment with RAL. The natural polymorphism M50I alone has no influence on strand transfer activity and viral RC. Relative to M50I alone, the combination of M50I and R263K increased resistance to DTG to 5.6-fold, but did not restore viral RC (Wares et al., 2014). Moreover, recent studies showed that the addition of E138K to R263K modestly increased resistance to DTG in cell culture (FC = 4.3) and slightly increased susceptibility to DTG in cell-free strand-transfer assay from FC = 3 to FC = 4.4. The combination of E138K and R263K decreased integrase strand-transfer activity to about 60% compared to the WT enzyme and failed to restore viral infectivity (∼twofold decrease) and RC (Mesplede et al., 2014).

Another common mutation for resistance to DTG that was selected in cell culture is G118R (Quashie et al., 2014). Viruses containing R263K or G118R, alone or in combination with secondary mutations, often severely impaired virus RC. This may be the reason that primary resistance to DTG is so rare in clinical studies performed to date.

The mutations at R263K, G118R, H51Y, and E138K in integrase have been shown to confer low-level resistance to DTG. A recent study tested the ability of viruses harboring DTG-resistant mutations at either R263K or G118R and/or H51Y in the development of other mutations associated with resistance to reverse transcriptase inhibitors such as lamivudine (3TC) or nevirapine (NVP) in cell culture. As expected, wild-type (WT) viruses developed M184V/I, typical mutations that confer resistance to 3TC, in the presence of this drug as early as 6 weeks. The virus with the H51Y mutation alone was not impaired in ability to generate M184V/I mutation under 3TC pressure. However, the virus harboring R263K was delayed in ability to generate M184V/I mutation by 2 weeks. Similarly, the V106A resistance mutation for NVP was detected in WT virus in the presence of NVP at 6 weeks, while the virus carrying R263K developed the V106A mutation between weeks 11 and 14, i.e., a delay of at least 5 weeks. The viruses containing a combination of H51Y/R263K developed the V106A resistance mutation at week 11, also a delay of 5 weeks. The G118R and H51Y/G118R containing viruses did not develop any relevant resistance mutations to 3TC or NVP until week 25, when Y181C that confers resistance to NVP was present. These results clearly show that the R263K or G118R mutations, alone or in combination with H51Y, can delay the emergence of mutations that confers resistance to each of NVP and 3TC. These delays might be most likely caused by the decreased viral RC associated with the DTG-resistance mutations (Oliveira et al., 2014).

In the development of HIV resistance to drugs, compensatory mutations in HIV often increase levels of drug resistance while simultaneously restoring impaired viral fitness to normal level. Of note, no such additional compensatory mutations have been identified for DTG, even in cell culture selection experiments, for more than 4 years (Wainberg et al., 2013; Mesplede and Wainberg, 2014). Interestingly, combinations of R263K with primary resistance mutations for RAL and EVG at positions 143 and 148 resulted in vastly diminished enzymatic activity that may be incompatible with viral survival, which helps to explain why R263K has never been observed in the clinic together with primary RAL or EVG mutations (Anstett et al., 2014).

So far, virological failure has not yet been reported in treatment-naive individuals receiving DTG-containing regimens in clinical practice (Mesplede and Wainberg, 2014). This may be partially explained by the fact that the presence of mutations that confer resistance to DTG can impair the ability of HIV to develop further resistance against 3TC and NVP. Thus, we conclude that a DTG-containing regimen may not lead to virological failure, even if the R263K mutation is present. This hypothesis will need to be verified by ultrasensitive sequencing of the integrase gene from residual plasma viral RNA or the DNA of lymphocytes of patients who have been successfully treated with DTG (Oliveira et al., 2014).

Since no treatment-naïve patient treated with DTG has yet developed resistance to this drug and since the R263K mutation confers only low-level resistance in cell culture, it was postulated that DTG may be useful in attempts to limit HIV persistence. To investigate the impact of the R263K mutation on HIV RC and the ability of HIV viruses to establish or be reactivated from latency and/or spread through cell-to-cell transmission, a series of constructs containing EGFP were created by site-directed mutagenesis, including pNL4-3-IRES-EGFP-INR_263K_, pNL4-3-IRES-EGFP-IN_E138K_, pNL4-3-IRES-EGFP-IN_E138K/R263K_. The relevant replication competent reporter viruses were produced and used to study the effects of R263K on HIV RC, the ability to establish latency and/or to spread through cell-to-cell transmission in Jurkat cells. As expected, the R263K mutation caused diminished RC and infection. However, the DTG-resistant viruses were still efficiently transmitted via cell-to-cell contacts, and were as likely to establish and be reactivated from latent infection as wild type viruses (Bastarache et al., 2014).

The integrase baseline sequence can affect each of these mutations in regard to susceptibility to DTG. One study showed that DTG was effective against RAL-resistant variants containing the N155H, Y143C, N155H/Y143C, and G140S/Q148H mutations in different cell types, including C8166, human primary monocyte derived macrophages (MDMs), peripheral blood mononuclear cells (PBMCs) (Pollicita et al., 2014). Similarly, it has been shown that DTG is effective against patient-derived RAL-resistant variants containing Y143 or N155 mutations in both macrophages and CD4+ T cells. Only Q148H/R-bearing variants showed a reduced level of susceptibility (FC 5.5–19; Canducci et al., 2013).

In one case, a RAL-resistant patient harboring N155H was switched to a DTG-containing regimen for about 10 months. The acquisition of mutations at T97A and E138K in integrase led to 37-fold resistance to DTG. After the use of DTG for 10 more months, the sequential acquisition of mutations at A49P, L68FL, and L234V led to further resistance to DTG (FC 63-fold). Of these, A49P and L234V are novel mutational pathways leading to emergent DTG resistance while on salvage therapy with the N155H mutation (Hardy et al., 2015).

It was also observed that the sequential acquisition of DTG resistance mutations was associated with significant deficits in viral replicative capacity (41%) relative to levels observed (101–187%) prior to treatment with DTG (Hardy et al., 2015). In another case, a patient harboring a virus with high level resistance to all reverse transcriptase and protease inhibitors and S119R, N155H, and E157Q mutations in integrase after RAL failure was treated with DTG and RAL was removed. One month later, viral load was undetectable and after 8 months, two new mutations T97A and S147G in integrase were detected (Carganico et al., 2014). The G118R mutation was detected in a RAL-failing patient harboring a subtype CRF02_AG virus and the F121Y mutation alongside other mutations was detected in a RAL-failing subtype B patient. Phenotypic analysis in cell culture showed that the G118R and F121Y mutations conferred broad cross-resistance to all three currently used INSTIs, with higher resistance observed with clinical isolates than with a NL43 backbone (Malet et al., 2014). These results suggest that DTG should be used with caution in INSTI salvage therapy for RAL failures. However, several INSTI-experienced patients with extensive resistance who were treated with DTG demonstrated excellent virological and immunological responses (Hofstra et al., 2014).

## Insights into Mechanisms of HIV Resistance to DTG

**FIGURE 1:**
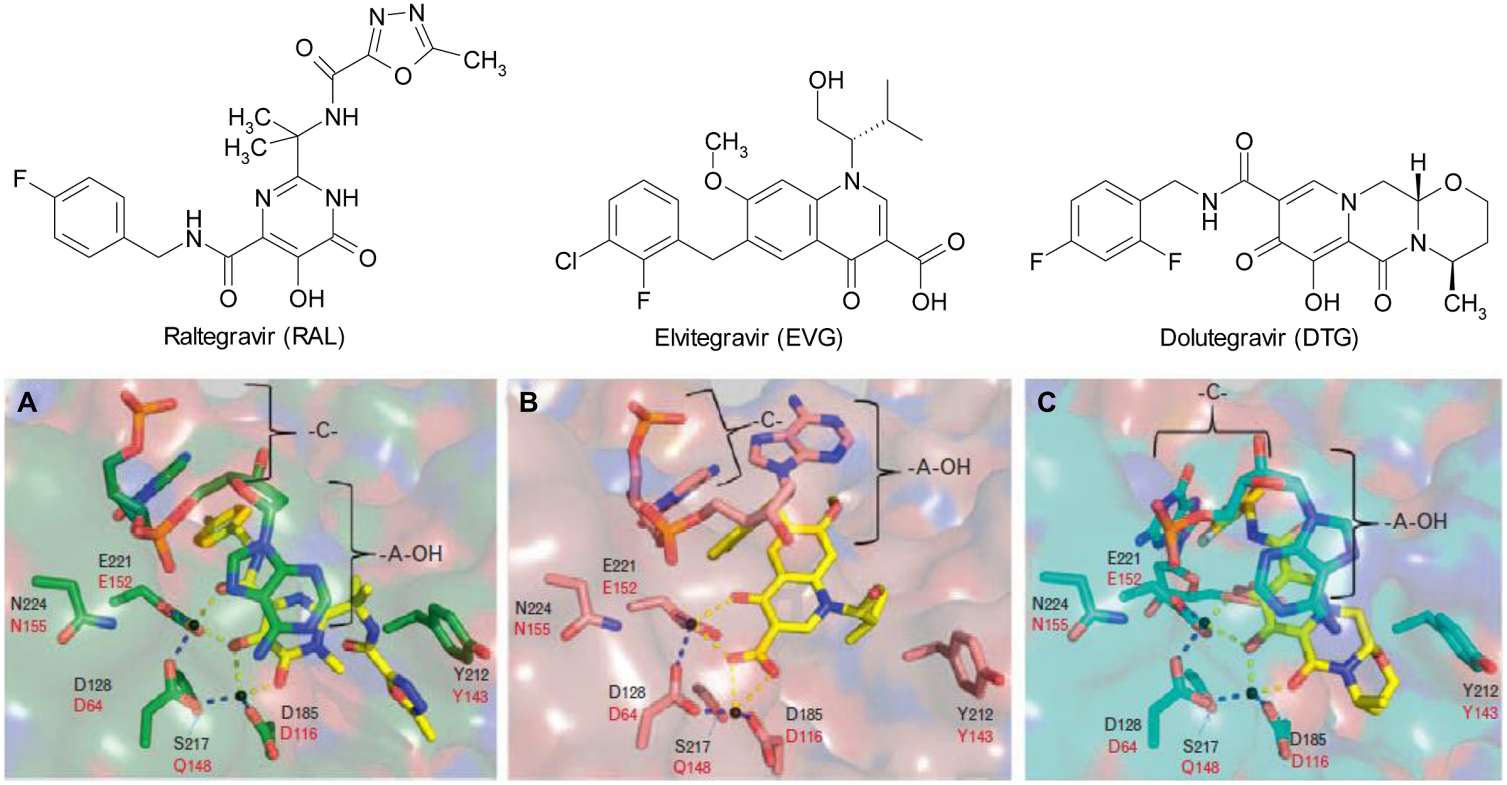
**Chemical structures of (A) raltegravir (RAL), (B) elvitegravir (EVG), and (C) dolutegravir (DTG) and their binding modes to the prototype foamy virus (PFV) integrase active site (Quashie et al., 2013b)**.

Recently, considerable progress has been made in studies on mechanisms of HIV resistance to DTG. It is widely accepted that the higher genetic barrier to resistance of DTG is due to its slow dissociation rate from integrase-DNA complexes in comparison to RAL and EVG (Hightower et al., 2011; Geretti et al., 2012; Wainberg et al., 2013). Studies on binding modes of all INSTIs to prototype foamy virus (PFV) intasomes, a surrogate for the HIV-1 intasome, have shown that an extended linker of DTG allows its difluorophenyl group to enter farther into the pocket within the integrase active site than other INSTIs and that DTG has the ability to adjust its structure and conformation in response to structural changes within the active sites of RAL- and EVG-resistant integrases, compared to RAL and EVG (**Figure 1**; Hare et al., 2011; Quashie et al., 2013b). Biochemical studies have provided further insight into the mechanism of resistance to DTG. It has been shown that the R263K mutation in integrase results in decreases in 3′-processing and strand transfer activities. Homology modeling of intasomes and strand transfer complexes from WT and R263K-containing integrases reveals altered interactions in integrase-DNA. In addition, an integrase-DNA binding assay showed that the R263K mutation decreased integrase-viral DNA binding (Quashie et al., 2012). Similarly, biochemical studies have shown that the G118R mutation in integrase greatly decreases strand transfer activity but does not affect 3′-processing activity (Quashie et al., 2013a).

The low levels of resistance to DTG conferred by mutations in integrase correlate with decrease in strand transfer activity and viral RC (**Table 2**). A recent study on homology modeling of constructed tetrameric HIV-1 intasome revealed the molecular mechanism of cross-resistance conferred by E138K/Q148K to RAL, EVG, and DTG. Using molecular dynamics simulation and residue interaction network (RIN) analysis, the work showed that P145 in the 140S loop (G140–G149) of the intasome has strong hydrophobic interactions with INSTIs and is involved in a conformational rearrangement at the active site of the HIV-1 intasome. Furthermore, a systematic RIN analysis demonstrated that communications between the residues in the resistance mutant are increased compared with those of the WT HIV-1 intasome. In addition, the chelating ability of the oxygen atoms in INSTIs (e.g., RAL and EVG) to Mg^2+^ in the active site of the resistant integrase was reduced due to conformational change and is most likely responsible for the cross-resistance (Xue et al., 2013). More recently, a computational analysis of G118R and F121Y mutation conferring high-level resistance to RAL, EVG, and DTG, showed that these substitutions were associated with reduced binding affinities to each of the INSTIs and with a decreased number of hydrogen bonds compared with the WT complexes (Malet et al., 2014). These findings provide valuable information on the mechanism of resistance to INSTIs and will be useful for structure-based design of novel INSTIs that may possess better resistance profile than currently available drugs.

## Perspectives

Currently, patients treated with DTG-containing regimens achieve high rate of efficient responsiveness up to 90%, though some patients have failed therapy. Interestingly, no resistance mutations to DTG in treatment-naive patients have yet been identified. Moreover, selection of resistance with DTG in cell culture has yielded only two mutations that confer low level resistance to this drug, in association with a decrease in viral RC. So far, no secondary compensatory mutations that might augment resistance and restore viral RC have been observed in selection experiments in cell culture over more than 4 years. These results may be explained by the fact that viruses containing DTG-resistance mutations are relatively replication impaired and may be unlikely to efficiently replicate in patients. If this is true, then the development of low-level resistance to DTG in first-line therapy might not have adverse clinical consequences and DTG could be used in Treatment as Prevention (TasP) protocols to reduce viral load on a population level, which will eventually result in diminished rates of HIV transmission (Mesplede et al., 2013; Wainberg et al., 2013).

In addition, it is conceivable that DTG could be employed as monotherapy in treatment-naive patients. In such a study, resistance mutations to DTG in both the RNA of patient plasma samples and the DNA of patient PBMCs would need to be intensively monitored by ultrasensitive sequencing methods. If the results are similar to those observed in the phase III clinical trials, i.e., an absence of resistance to DTG, this would help to validate the hypothesis outlined above. Could a number of cycles of DTG monotherapy conceivably convert all the HIV, even that in latent reservoirs, into replication-impaired forms? Perhaps such an approach could lead to a functional cure of HIV disease if all residual viruses were significantly impaired in viral replication and if further compensatory mutations were unable to develop. This concept could first be studied in animal models such as rhesus macaques that are infected by simian immunodeficiency virus (SIV) or humanized mice that are infected by HIV (Wainberg et al., 2013).

## Conclusion and Future Directions

Although INSTIs are highly potent drugs, HIV may still be able to develop mutations that confer resistance against all currently available ARVs. Thus, further characterization of the resistance profile of DTG, both *in vitro* and in the clinic, is important. More sensitive assays are important, such as next-generation sequencing for the detection of low-level viremia and minority resistance variants. Non-human primate models are important tools with which to study issues of drug resistance as well as the persistence and transmission of drug-resistant viruses (Hassounah et al., 2014; Wares et al., 2015). Moreover, the development of new classes of anti-HIV drugs with high resistance barriers that have no cross-resistance with current drug classes is still needed (White et al., 2014). Recently, it has been reported that a compound similar to DTG, termed GSK1265744, can act as an INSTI on a once-daily basis and that this drug possesses a distinct resistance profile compared with the earlier INSTIs, RAL, and EVG (Yoshinaga et al., 2015). This provides hope for the future of HIV prevention and treatment.

## Key Concepts

Antiretroviral therapy: a regimen that consists of a combination of at least three antiretroviral drugs to maximally suppress HIV replication and stop the progression of HIV disease.

HIV drug resistance: the presence of HIV mutations that reduce drug susceptibility compared with WT viruses.

Integrase strand transfer inhibitor: compounds that block the strand transfer reaction of HIV integrase to prevent HIV replication.

Viral fitness: the ability of a virus to reproduce itself in host cells.

HIV RC: a measurement of the virus’s fitness.

Virologic failure: the inability to achieve or maintain suppression of viral replication to level less than 50 copies viral RNA/ml of plasma.

## Conflict of Interest Statement

The Editor Rongtuan Lin declares that, despite being affiliated to the same institution as the authors Mark Wainberg and Yingshan Han, the review process was handled objectively and no conflict of interest exists. The authors declare that the research was conducted in the absence of any commercial or financial relationships that could be construed as a potential conflict of interest.
